# “Never Trust the Skin”: A Rationale for Using Polydioxanone Internal Support Matrix to Minimize Scarring in Primary Mastopexy-Augmentation—An Observational Study

**DOI:** 10.1093/asjof/ojac048

**Published:** 2022-05-19

**Authors:** Julia A Chiemi, Shahrooz Sean Kelishadi

## Abstract

**Background:**

The process of scar formation is complex and multi-factorial. Basic plastic surgery tenets focus on tension-free techniques to optimize aesthetic outcomes and minimize scarring.

**Objectives:**

Prophylactic use of a polydioxanone (PDO) internal support matrix in cosmetic mastopexy-augmentation to decrease scar burden has never before been described.

**Methods:**

A high volume (n = 41) single-surgeon mastopexy-augmentation experience (S.S.K.) followed scar quality in consecutive cases from June 2020 to July 2021. A minimum of 6 months of postoperative evaluation was required to assess scar quality. Fitzpatrick scores were also evaluated and compared. All surgeries in this study were performed in the dual plane using silicone gel implants, a superior or superomedial dermal pedicle blood supply, and a wise-pattern or vertical scar. Scar quality was evaluated by photography and scored according to an internally developed scar quality scale.

**Results:**

There have been no cases of hypertrophic or keloid scarring. All patients receiving mastopexy-augmentation with prophylactic PDO mesh have a favorable appearance with fine line scars, and the mean scar quality scale score across the cohort was 4.341/5. The mean Fitzpatrick scale score across the cohort was 2.97, and, of the patients who scored a 5 on the scar quality index, the mean Fitzpatrick scale score was 3.545.

**Conclusions:**

Prophylactic use of PDO internal support matrix in silicone gel mastopexy-augmentation offers further protection against poor scarring in patients across the Fitzpatrick scale, with varying degrees of skin quality, and across medium to high-volume implant augmentations. Patients who received PDO prophylaxis demonstrated a better-than-average scar appearance.

**Level of Evidence: 4:**

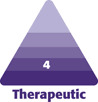

By far, the most notorious part of a mastopexy procedure is the associated scar. There are patients who are clear candidates for an augmentation lift but fear developing a prominent, thick, or unsightly vertical scar; they often seek to avoid this necessary part of their procedure at all costs. Frequently, these patients are disappointed by their outcomes after undergoing an augmentation alone when their ptosis remains unimproved or their breast aesthetics are worsened in appearance by an implant. These patients either remain unhappy with their breast augmentation or end up receiving a separate mastopexy down the line.

The process of scar formation is complex and multifactorial. Though much of scar healing and appearance is dependent on a patient’s genetics, connective tissue health, and compliance with postoperative instructions (such as avoiding ultraviolet exposure and consistent scar treatment usage when prescribed), surgeon technique is undoubtedly crucial to an aesthetic result.^1^ Basic plastic surgery tenets focus on tension-free techniques to optimize aesthetic outcomes and minimize scarring. This has commonly come to mean utilizing multilayered suture closure and a thoughtful selection of implant size. In patients presenting with thin, poorly toned skin or inadequate soft tissue stores, more support may be necessary. Additional soft tissue reinforcement through the use of internal support matrices (hereinafter referred to as “mesh”) was hypothesized to decrease the pressure of the implant volume on the mastopexy flaps, which could contribute to better scar formation.

Mesh has been used in breast surgery for reinforcement of soft tissue and is available in a variety of materials.^2,3^ DuraSorb (SIA, Chicago, IL) polydioxanone (PDO) mesh (a synthetic absorbable polymer similar to polydioxanone [PDS] suture) was cleared by the FDA in 2018 for soft tissue support.^4^ Its early tissue integration and absorption profile were hypothesized to be an excellent option for decreasing tension on the mastopexy incision during the crucial first months of wound healing. The prophylactic use of a PDO internal support matrix in cosmetic mastopexy-augmentation to decrease scar burden has never before been described.

## METHODS

A retrospective cohort analysis was conducted using data collected from 41 consecutive primary mastopexy-augmentation surgeries performed between September 2020 and July 2021 utilizing bilateral smooth silicone gel breast implants plus PDO internal support matrix. A minimum of 3 months of postoperative evaluation was required to assess scar quality. The surgeries were performed by the senior author (S.S.K.) in Newport Beach, California. All mastopexy augmentations in this study were performed in the dual plane using a superior or superomedial dermal pedicle blood supply and with a wise-pattern or vertical mastopexy scar. Patients with silicone gel breast implants of all sizes with a smooth, round shell were included in this study, ranging from 350 to 700 ccs. The patients were similarly healthy, and all patients were female ranging from 19 to 64 years of age, with the average age in the cohort being 33.6 years. Written consent was provided, by which the patients agreed to the use and analysis of their data.

Three-layered suture closure was used for all cases (fascial layer to cover the implant, deep dermis, and a subcuticular layer). Drains were never used. Patients consented to retrospective and prospective review of their case data at the time of their preoperative appointment.

While developing the dual-plane pockets, the monofilament mesh was removed from its sterile packaging and soaked in a triple antibiotic irrigation solution consisting of 50,000 units of bacitracin, 1 g of cefazolin, 80 mg of gentamicin, 1 liter of normal saline, and 1 liter of povidone-iodine solution. After dissection, the mesh was removed from the solution and cut in half. Each half was oriented such that the smooth surface would be facing toward the patient’s breast implants and the rough surface toward the breast tissue. The mesh was then contoured to the confines of the breast implant pocket and inset to the periosteum of the rib and the Scarpa fascia using 2-0 Vicryl (Ethicon, Raritan, NJ) sutures in an interrupted fashion along its inferior edge, going from medial to lateral along the inframammary fold border. In a pure vertical mastopexy-augmentation, the breast implants were inserted first using an introduction sleeve, and then the mesh was inset as described above, with the smooth surface of the mesh placed against the breast implant and its rough surface toward the breast tissue. The superior border of the mesh covered at least the lower half of the breast implant and did not need any sutures to suspend its superior border. 2-0 Vicryl sutures in running simple and locking fashion were used to reapproximate the fascia of the breast gland over the implant and mesh. In cases where a wise-pattern mastopexy-augmentation was performed, the implants were placed, and the breast fascia was then closed to have total implant coverage. Then, the mastopexy was carried out, flaps were elevated, and the mesh was sewn outside the breast implant pocket, but still with its most inferior border securing the inframammary fold border. While the mesh was still oriented with the smooth surface toward the breast implant and its rough surface toward the mastopexy flaps, the main difference was that its superior border and any dead space were quilted with light 2-0 Vicryl interrupted sutures tacked down to the underlying soft tissue being sure not to go too deep and inadvertently puncture the underlying implant. If the patient’s soft tissue was very thin, the mesh would sometimes be placed inside of the breast implant pocket similar to what is done in a vertical mastopexy in order to have more tissue coverage over the mesh. Otherwise, the senior author would perform a routine mastopexy with tailor-tacking, marking, release of staples, de-epithelialization of the intended blood supplying pedicle, excision of excess skin and subcutaneous fat, debulking of excess lower pole breast volume, and 3-layered suture closure.

**Figure 1.**
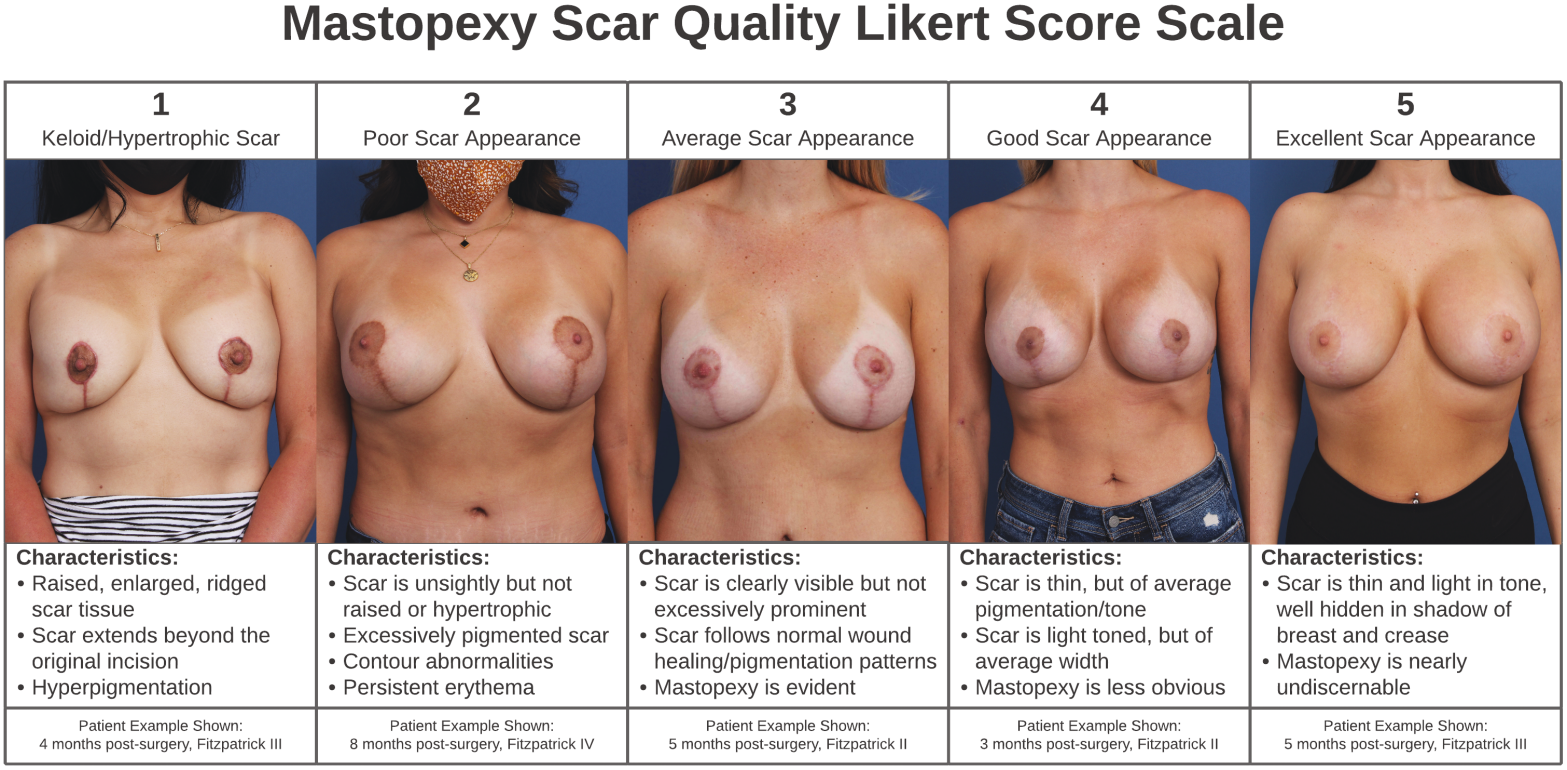
The 5-category inventory used to scale wise-pattern mastopexy scar appearance across the study cohort displays examples of the prototypical scare for each score, categorical criteria, and data on example photographs shown. This reference was used to create a calibrated scale against which the cohort with mesh placement could be compared with gauge scar appearance.

Patients were monitored with the typical in-person follow-up schedule of 1 week postsurgery, 1 month postsurgery, 3 months postsurgery, 6 months postsurgery, and yearly follow-up appointments for each subsequent anniversary. Patient photographs were reviewed at a minimum of 6 months follow-up and beyond to assess final scar quality. Patients were given a Fitzpatrick scale score as gauged by their natural skin tone without sun exposure/artificial tanning and natural hair color^5^ (Table 1). Scar quality/appearance was evaluated by photography and then scored by an independent observer on an internally developed Likert-type scale (Figure 1).

**Table 1.** The Fitzpatrick Skin Phototype Scale

Fitzpatrick skin phototype	Reaction to sun exposure	Complexion
I	Always burns, never tans	Very fair
II	Burns easily, burns develop into a light tan	Fair to light
III	Burns a moderate amount, burns develop into a light tan	Light to medium
IV	Burns a minimal amount, develops a moderate tan	Moderately dark
V	Does not burn, develops dark tan	Dark
VI	Does not burn or change in appearance	Very dark

Table adapted from Ellers et al. 2013.^5^

## RESULTS

All patients who received primary mastopexy-augmentation with the prophylactic placement of PDO internal support matrix had a favorable result with fine line scars. There have been no cases of hypertrophic or keloid scarring.

**Figure 2.**
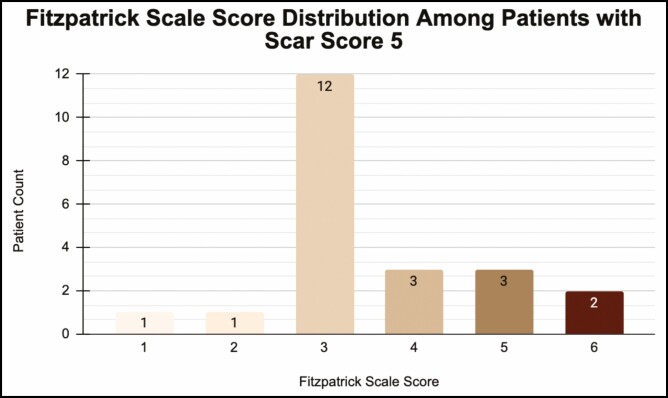
Distribution of Fitzpatrick scale scores across the study cohort.

**Figure 3.**
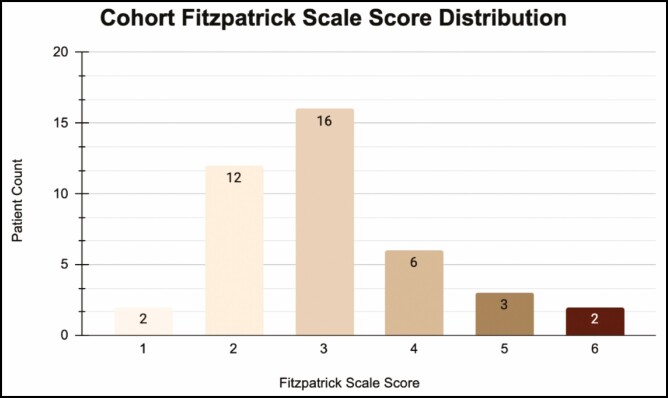
Distribution of Fitzpatrick scale scores across the patient group who scored 5 on the Mastopexy Scar Quality Likert score scale.

The average follow-up time was 8.4 months. The mean scar quality scaled score across the cohort was 4.341 (Good-Excellent). No patients scored below 3 on the scar quality index (Table 2). The mean Fitzpatrick scale score across the cohort was 2.97 (Figure 2). Of the patients who scored a 5 on the scar quality index, the mean Fitzpatrick scale score was 3.545 (Figure 3).

**Table 2.** Distribution of Mastopexy Scar Quality Likert Scores Across the Study Cohort

Score	No. of patients
1 Keloid/hypertrophic	0
2 Poor	0
3 Average	8
4 Good	11
5 Excellent	22

Wise-pattern scar appearance scores in primary mastopexy augmentations with prophylactic polydioxanone mesh placement (n = 40). Final scar scores were coded by an independent observer at uniform follow-up postsurgery.

## DISCUSSION

Scar formation is governed by a multitude of factors and is often a major concern for patients undergoing elective aesthetic surgery. As such, scar management has become a multi-billion dollar industry in the United States,^6^ yet we lack a conclusive mechanism of fibrotic hypertrophic scar formation or a standardized protocol for plastic surgeons to reduce and manage scarring in patients of all ethnicities. Current research on aberrant wound healing points to an exaggerated immune response, epithelial abnormalities, and the individual’s connective tissue tensile strength as the main contributors to poor scarring results.^7^ Scarring is known to be at least partially driven by genetics; Asian skin has a tendency toward hyperpigmentation with injury.^8^ Studies on melanocyte proliferation have also shown darker-skinned patients (such as Black, Hispanic, and some Asian populations) to be more susceptible to keloid scarring than Caucasians.^9^ Given this wide variation in scarring patterns, the authors sought to investigate a prophylactic method that would serve a protective effect in all demographics and provide a more predictable healing trajectory for mastopexy patients.

Plastic surgeons have long focused on using tension-free techniques to minimize scarring and wound dehiscence. The mastopexy incision often poses a challenge to proper wound healing, as the tight closure over an implant after the removal of excess skin in the vertical and/or horizontal aspect creates an incision under considerable tension. This strain sometimes manifests in superficial dehiscences or pinhole openings of the wound at the T-junction which typically close on their own without complication but can result in worsened scar appearance in their place. Given these trends, we hypothesized that lowering the internal tension force on the mastopexy scar will result in improved scar appearance during healing and beyond. By providing additional soft tissue support, it was predicted that the mesh would act as a scaffolding to reduce pressure on the incision closure, lowering the risk of the scar stretching or widening and also promoting connective tissue health. These combined benefits from the mesh would create an optimal environment for the best scarring outcome; thus, the long-term appearance of the mastopexy scar would largely be determined by the internal mechanisms of tissue repair during the first crucial weeks of healing.

The authors realize that, in most cases, natural physiology dictates that scars tend to improve in appearance over time, usually over 1 to 2 years. At 6 months postsurgery, they tend to be more noticeable, and this is why many of the patients in this study were assessed for scar quality at less than 1 year postsurgery. In our findings, we discovered that scars with mesh at 6 months often appeared similar to scars we had seen without mesh at longer-term stages of healing. 

**Figure 4.**
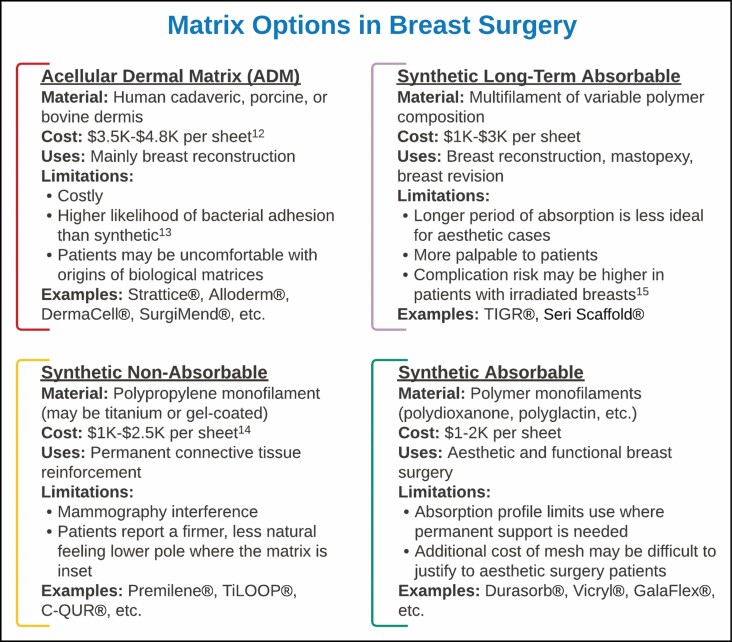
A summary of the material, monetary cost, benefit, and specific limitation profiles of the 4 most commonly used mesh categories in reconstructive and cosmetic breast surgery. “Cost” represents an estimated range based on current market prices and may vary by region and manufacturer.

A careful rationale underlies our original interest in mesh as a prophylactic measure in cosmetic breast cases, which, as an application, has been virtually unheard of. Mesh exists in a variety of biological and synthetic options on the market and has been utilized for years in breast surgeries but up until recently was used almost exclusively in reconstructive rather than aesthetic cases.^10^ Biological meshes or acellular dermal matrices (ADMs) are derived from human cadaveric, porcine, or bovine dermis that has been aseptically processed to remove cells and preserve an extracellular matrix scaffolding.^11^ These meshes are effective, yet are costly and carry additional risks with their implantation including bacterial infection, seroma, and other postoperative complications.^12^ Synthetic meshes were created as a more cost-effective and inert option for reinforcement and also exist in a variety of materials and absorption profiles (Figure 4).^12-15^ Synthetic mesh options range from permanent, non-absorbable matrices^13^ such as titanium-coated (TiLoop, FM Medical, Carlsbad, CA) or gel-coated (C-QUR, Atrium Medical, Merrimack, NH) polypropylene, slow-absorbing meshes made of polyethylene or filaments with mixed absorption profiles^14^ (TIGR, Novus Scientific, Uppsala, SWE and Proflex Omnia, Clovis, CA), or fully absorbable synthetic matrices like the DuraSorb polydioxanone matrix used in this study.^15^ Synthetic meshes all carry less cost than ADMs but still vary widely in cost by region and manufacturer. Of the above categories, synthetic fast-absorbing matrices tend to be the least expensive option but are still underutilized in cosmetic cases. Mesh is still viewed by most aesthetic plastic surgeons as a product to be used in “bail-out” revision cases rather than a tool in their armamentarium to provide durable, stable long-term results to cosmetic patients. Thus, mesh use in primary augmentations and primary mastopexy-augmentations is relatively novel and not yet described in research.

When designing patient selection criteria for this study cohort in order to best assess mesh’s potential in a prophylactic application, the authors considered a variety of factors before deciding to use DuraSorb mesh in all primary mastopexy-augmentation patients during the study time frame. Though we originally considered using mesh only in patients receiving high-volume implants (≤450 cc) with their mastopexy, mesh was ultimately extended to use in all mastopexy-augmentation patients of S.S.K. This was so that we could fully consider the protective benefit, if any, of mesh against poor mastopexy scarring regardless of selected implant size (which is governed by both patient anatomy and individual taste). Limiting mesh use to only high-volume implant cases may have unduly limited the study’s demographic. Additionally, there are little data to suggest that the size (in cc’s) of the implant used in mastopexy-augmentation has a significant impact on scar appearance, which appears to be more dependent on surgeon technique, tension-free closure, and incision aftercare. Tight closure of a mastopexy-augmentation with any volume of implant underneath contributes additional tension to the wound that is not present in a mastopexy without implants. Further research is needed before concluding that large implants are a risk factor in unfavorable mastopexy scarring. Thus, we decided to use mesh in all mastopexy-augmentation patients regardless of the size of the smooth round implant they were receiving. The average implant volume used across the cohort was 516.9 cc’s.

Another reason for the broad cohort selection was the diverse patient population served by the practice and in Southern California. Considering the aforementioned genetic and phenotypic associations with poor scarring outcomes, black, Hispanic, and Asian patients were hypothesized to be at a higher risk for scarring than Caucasian patients, and we wished to capture as many of these high-risk patients as possible within the study set in hopes of improving their aesthetic outcomes. S.S.K. serves a heterogeneous patient population in Orange County, California—many patients express concern over prominent mastopexy scars at their consultations, citing a variety of factors including revealing clothing trends, a subjective importance placed on their breast’s appearance both in and out of clothing, and a general Californian cultural focus on “looking good.” Therefore, all primary mastopexy-augmentation patients were deemed candidates for this prophylactic intervention and were counseled extensively during their 1-hour consultation appointments on their options. Patients were educated on the PDO mesh to be used, its novel nature in this application of breast surgery, its costs and benefits, and its hypothesized protective effect against poor scarring results that was being studied. All patients who were given the option to receive mesh implantation as part of their surgery opted to receive it, with no patients asking to receive mastopexy-augmentation alone after counseling.

Ultimately, it was our observation that prophylactic placement of PDO mesh in the lower pole of the breast pocket provided good to excellent protective results against scarring throughout the cohort. It is our judgment that this added soft tissue support was effective at taking pressure off the wise-pattern incisions over the first 3 months of its absorption profile, providing tension-free healing that allowed for a better-than-average scar appearance early in the healing process. These results also held up well across the Fitzpatrick scale, indicating that this prophylactic measure does not only benefit Caucasian and light-skinned patients who might have scarred well regardless, but even more so in patients with high Fitzpatrick scores. For example, one of the darkest-complexioned patients (Fitzpatrick VI) in the study cohort presented with bilateral striae to the breasts after pregnancy and overall poor skin quality that we noted as a risk factor for mastopexy scarring before surgery. This patient’s results scored a 5-Excellent on the Likert scale measure. We believe that the mesh’s soft tissue reinforcement in this patient was the major contributing factor in her optimal aesthetic results along with others with naturally darker skin tones.

**Figure 5.**
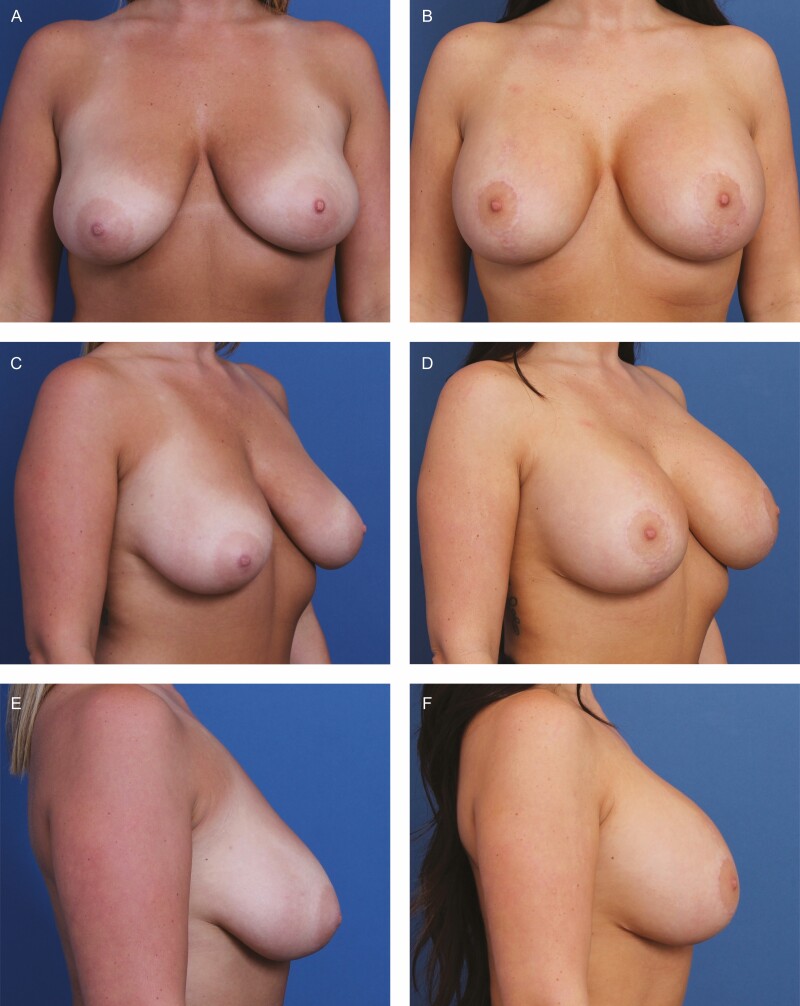
A 31-year-old female patient shown 12 months after primary mastopexy-augmentation using Sientra HSC+ High Profile Round Smooth Implants, 470 cc, plus DuraSorb mesh (SIA, Chicago, IL). The patient scored a 5 on the Scar Quality Likert scale and is a Fitzpatrick III. Frontal, three-quarter, and lateral views shown at (A, C, E) preop and at (B, D, F) 5-month follow-up.

**Figure 6.**
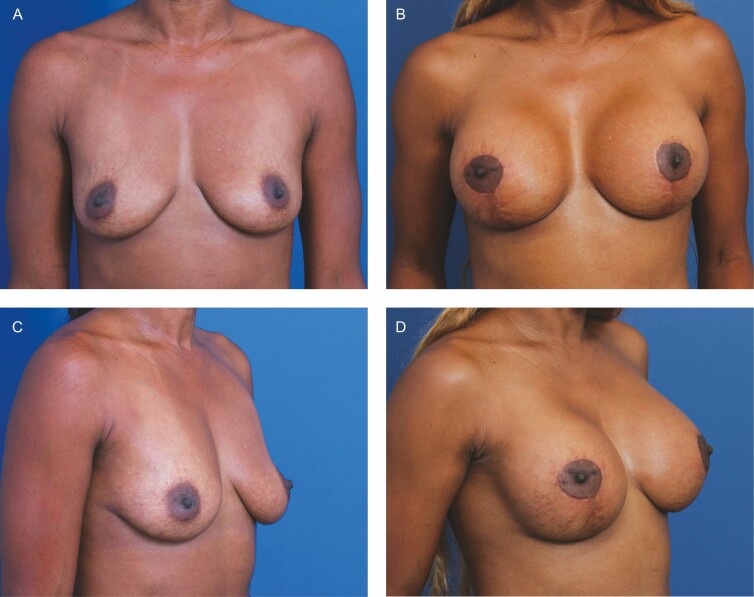
A 29-year-old female patient shown 6 months after primary mastopexy-augmentation using Allergan Natrelle Inspira SoftTouch SSF Smooth Round Implants, 450 cc (Irvine, CA), plus DuraSorb mesh (SIA, Chicago, IL). The patient scored a 4 on the Scar Quality Likert scale and is a Fitzpatrick VI. Frontal, three-quarter, and lateral views shown at (A, C) preop and at (B, D) 5-month follow-up.

**Figure 7.**
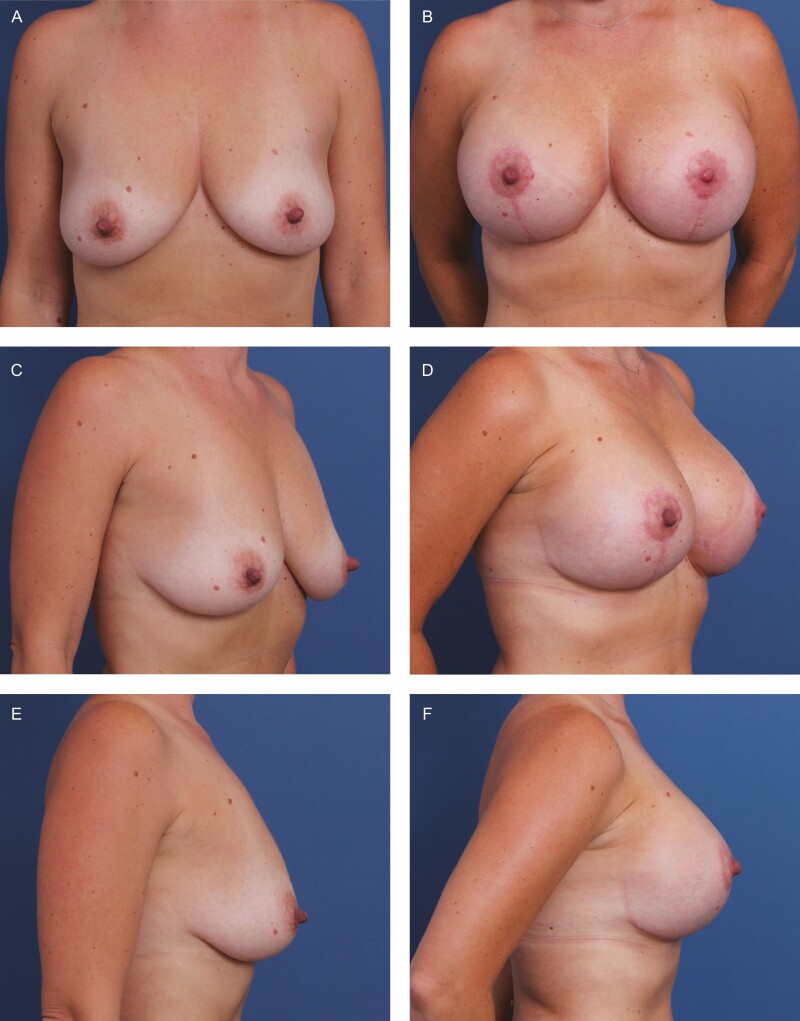
A 54-year-old female patient shown 6 months after primary mastopexy-augmentation using Sientra HSC+ High Profile Round Smooth Implants, 505 cc (Santa Barbara, CA), plus DuraSorb mesh (SIA, Chicago, IL). The patient scored a 4 on the Scar Quality Likert scale and is a Fitzpatrick II. Frontal, three-quarter, and lateral views shown at (A, C, E) preop and at (B, D, F) 5-month follow-up.

**Figure 8.**
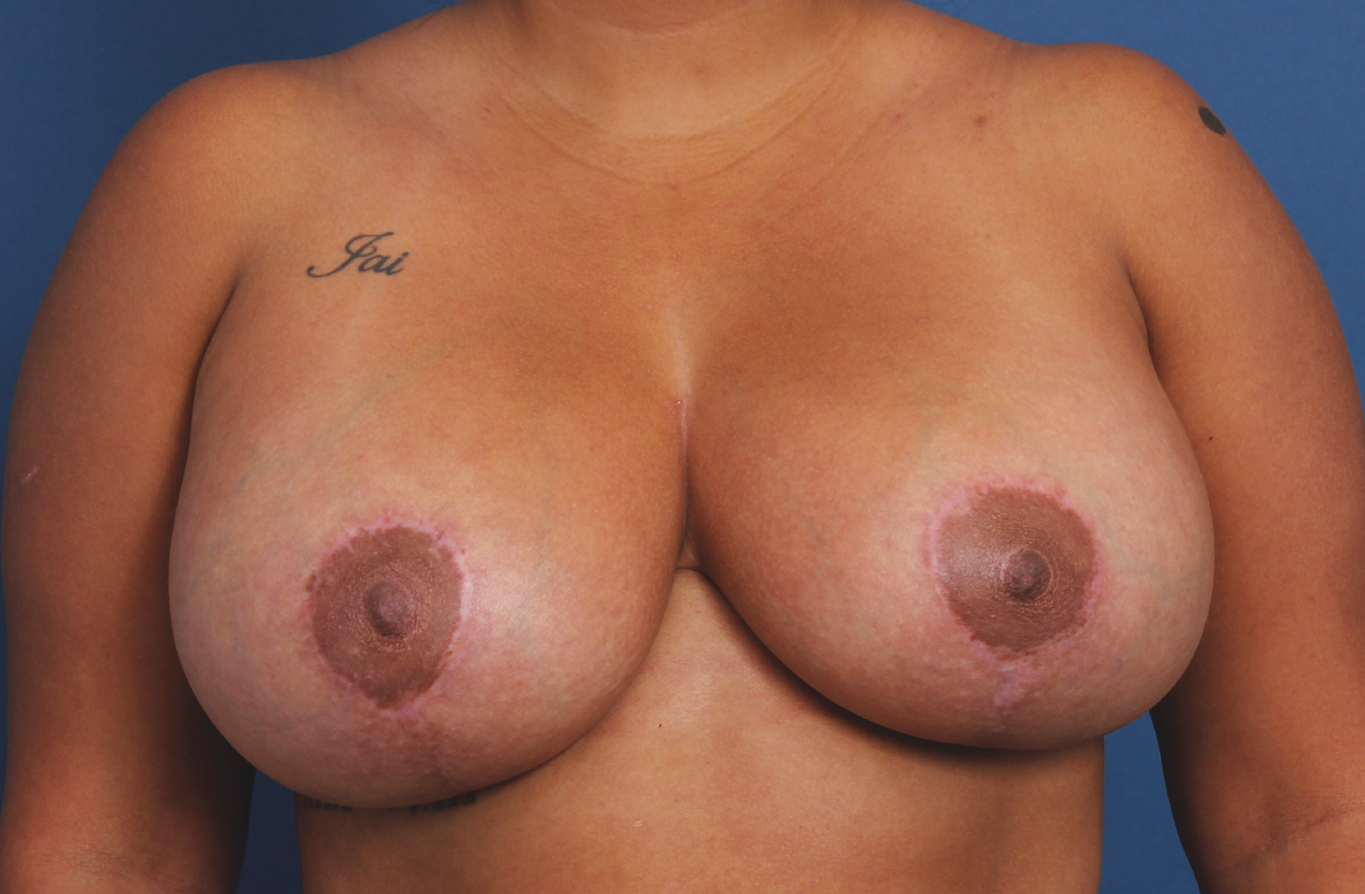
A 34-year-old female patient shown 6 months after primary mastopexy-augmentation using Sientra HSC+ High Profile Round Smooth Implants, 505 cc, plus DuraSorb mesh (SIA, Chicago, IL). The patient scored a 5 on the Scar Quality Likert scale and is a Fitzpatrick V.

**Figure 9.**
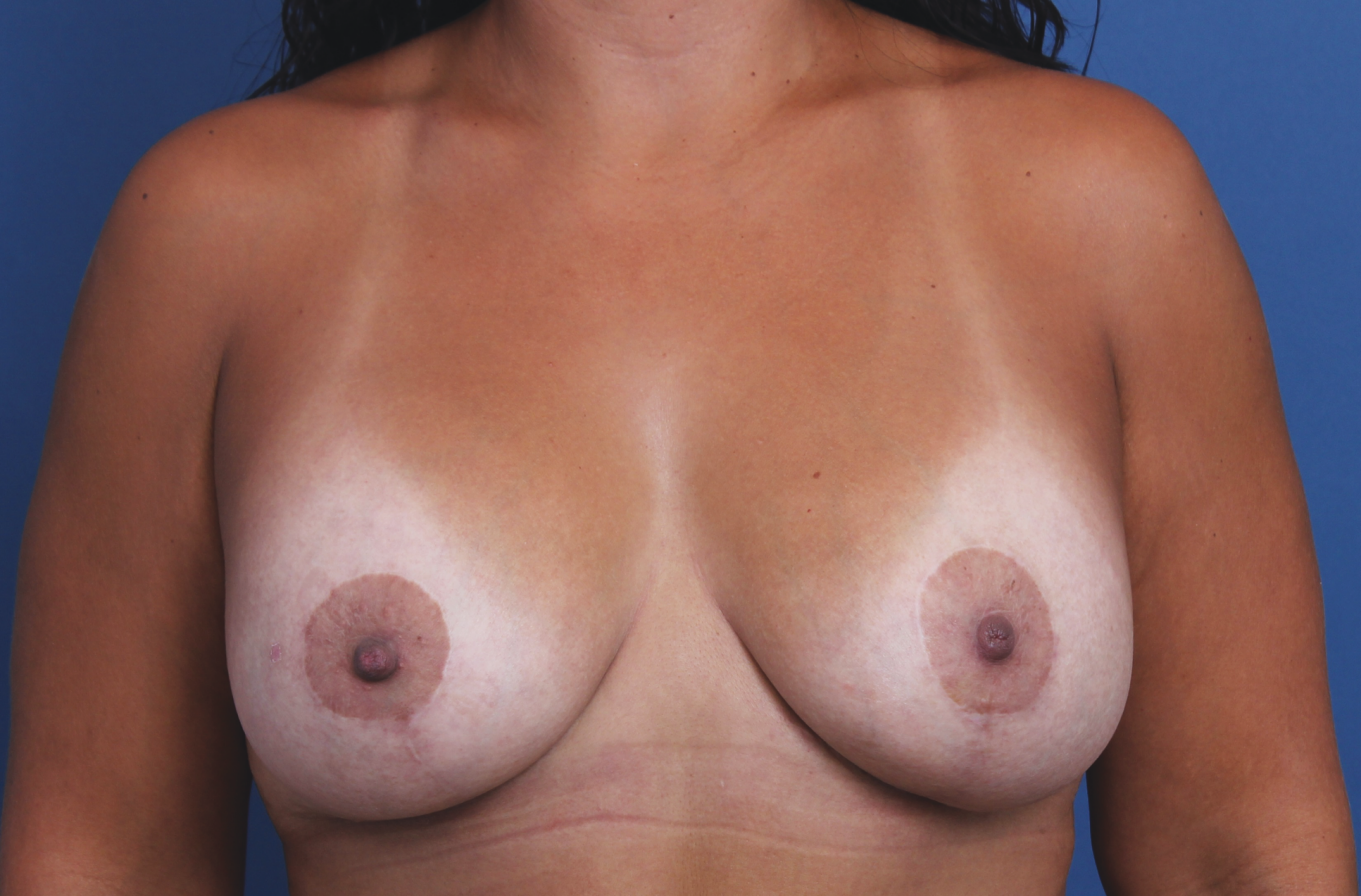
A 37-year-old female patient shown 10 months after primary mastopexy-augmentation using Allergan Natrelle Inspira SoftTouch SSF Smooth Round Implants, 485 cc (Irvine, CA), plus DuraSorb mesh (SIA, Chicago, IL). The patient scored a 5 on the Scar Quality Likert scale and is a Fitzpatrick III. This patient was a massive weight loss patient and had very weak soft tissue stores.

**Figure 10.**
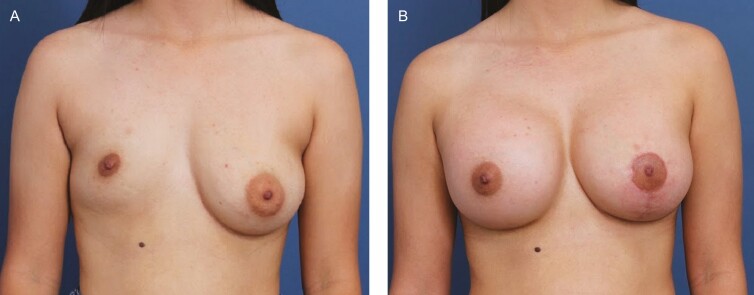
A 19-year-old female patient shown 8 months after primary bilateral augmentation and left mastopexy using Allergan Natrelle Inspira SoftTouch Smooth Round Implants, R SSF 450 cc and L SSM 360 cc (Irvine, CA), plus DuraSorb mesh (SIA, Chicago, IL). This patient scored a 4 on the Scar Quality Likert scale and is a Fitzpatrick IV. Frontal view shown at (A) preop and at (B) 8-month follow-up.

This was a small study looking at the impact of mesh on a subset of patients at our practice (Figures 5-10). The limitations of this study were the use of a nonvalidated scale for scar assessment (the study scar scale was internally developed to help guide patient care, but since its development was never published, it is non-validated in the sci, evaluation of the complete vertical scar with the patient in an erect position with their hands down, and the use of a single independent observer. Mesh appeared to have a positive effect and had no negative effect on scar quality or healing. Many of the patients in this cohort received aggressive mastopexies with implants above 500 mL and did well. The additional soft tissue reinforcement provided by mesh allowed us to feel more confident using larger-volume implants in patients receiving a mastopexy, allowing us to meet patients’ aesthetic expectations. While this study only examined primary mastopexy-augmentations, the authors of this study were also utilizing PDO mesh in breast augmentations^16^ and breast revisional cases as well and saw excellent results and improved scarring in females who had already undergone previous mastopexy-augmentations with different surgeons. While outside the scope of this paper, further cohort or case studies may be warranted to examine the use of mesh in these patient groups as well as to improve the quality of scarring and durability of results.

## CONCLUSIONS

This prospective cohort study found that the prophylactic use of PDO internal support matrix in silicone gel mastopexy-augmentation offers further protection against poor scarring. The protective effects of mesh were seen in patients of fair to dark skin tones, with varying degrees of skin quality, patients with and without children, and patients receiving lower and high-volume silicone gel implants. We, therefore, conclude that the prophylactic placement of PDO mesh is a safe, cost-effective, and multi-beneficial technique in primary mastopexy-augmentations for patients desiring optimal scarring, durable pocket control, and excellent long-term aesthetic results.
